# Characterizations of how species mediate ecosystem properties require more comprehensive functional effect descriptors

**DOI:** 10.1038/srep06463

**Published:** 2014-09-24

**Authors:** R. Hale, M. N. Mavrogordato, T. J. Tolhurst, M. Solan

**Affiliations:** 1 University of East Anglia, School of Environmental Science, Norwich, NR4 7TJ, UK; 2 University of Southampton, Engineering and the Environment, Highfield, Southampton, SO17 1BJ, UK; 3 Ocean and Earth Science, National Oceanography Centre, Southampton, University of Southampton, Waterfront Campus, European Way, Southampton, SO14 3ZH, UK

## Abstract

The importance of individual species in mediating ecosystem process and functioning is generally accepted, but categorical descriptors that summarize species-specific contributions to ecosystems tend to reference a limited number of biological traits and underestimate the importance of how organisms interact with their environment. Here, we show how three functionally contrasting sediment-dwelling marine invertebrates affect fluid and particle transport - important processes in mediating nutrient cycling - and use high-resolution reconstructions of burrow geometry to determine the extent and nature of biogenic modification. We find that individual functional effect descriptors fall short of being able to adequately characterize how species mediate the stocks and flows of important ecosystem properties and that, in contrary to common practice and understanding, they are not substitutable with one another because they emphasize different aspects of species activity and behavior. When information derived from these metrics is combined with knowledge of how species behave and modify their environment, however, detailed mechanistic information emerges that increases the likelihood that a species functional standing will be appropriately summarized. Our study provides evidence that more comprehensive functional effect descriptors are required if they are to be of value to those tasked with projecting how altered biodiversity will influence future ecosystems.

Changes in the structure and composition of communities driven by environmental variability and anthropogenic forcing have concomitant effects on biogeochemical cycles and other important ecosystem processes and functions of global importance^1^, but the direction and magnitude of response is highly dependent on the functional contributions of the modified extant community^2^. Changes to the portfolio of functional effect traits within an assemblage can be more important than alterations to species richness^3^, and has renewed interest in developing approaches^4^ that relate observed changes in ecosystem properties to the functional attributes of species. This is appealing because consideration of functional effect traits can offer mechanistic explanations of how changes in the biotic community result in transformations in functioning at habitat or ecosystem scales^5^, which is not always possible when relating changes in function to more abstract or less directly connected concepts, such as species relatedness or altered phylogenetic diversity^6^.

When assigning individual species to functional effect groupings, a fundamental requirement is that the relevant aspects of how a species mediates specific ecosystem properties have been accounted for and characterized. In order to be useful, summary functional effect descriptors need to provide the most relevant representation possible and be sufficiently comprehensive so that they provide acceptable explanatory power for observed species contributions to ecosystem function^7^. However, at least for marine benthic communities, this requirement is seldom fulfilled or tested^8^,^9^,^10^, and complementary functional descriptors are rarely simultaneously presented^11^ or combined to improve fit^2^. Furthermore, in practice, assigning individual species to specific functional effect groupings tends to be based on perceived contributions to functioning *per se*^12^, rather than on direct measurements of specific functions obtained in the presence of known species^13^. A fundamental problem is that the importance of organism-sediment interactions in determining how traits are expressed and mediate function is largely ignored^11^,^14^; in particular, the effects of biogenic structures, such as tubes, burrows and mounds, have not been considered as useful descriptors when determining the full extent of a species influence on ecosystem properties. Instead, functional effect groupings have become synonymous with trophic feeding position, morphological or physiological characteristics, or descriptions of specific sets of behavior^15^, restricting interpretative capacity of how species and assemblages mediate ecosystem process and functioning to a subset of the total inventory of effect parameters^16^. Rather than diversify the number of available functional effect parameters to address this shortcoming, recent efforts have focused on the details of how species contributions alter in relation to changes in context^5^,^17^,^18^,^19^. Whilst these have been informative, the mainstay of these studies perpetuates a limited view of what constitutes the functional role of a species. Any derived indices or coefficients, therefore, typically offer low levels of explanatory power and are of limited value in explaining the mechanistic basis for biodiversity-function relations^20^.

Despite evidence of their pivotal influence in shaping ecosystem properties^21^,^22^,^23^, the modifying effects of biogenic structures and species behaviour (e.g. burrows, mounds, pits, tubes, irrigation and/or feeding behaviour^24^) remain a less prominent feature of functional classifications^15^. Whilst context-dependent changes in species behaviour (e.g. in relation to food supply^18^, tidal periodicity^25^, temperature^26^ or photoperiod^27^) and key features of biogenic structures, including length, diameter, volume and configuration, have been described in detail for individual species^28^,^29^,^30^ and communities^31^, there is a paucity of information on whether such information is of utility in distinguishing the functional standing of individual species^32^,^33^,^34^. Here, we investigate the functional effect of three co-occurring species of benthic invertebrates, alone and in mixture, using a combination of high-resolution sediment profile imaging and computerized tomography that, together, allow multiple components of particle and fluid transport to be assessed alongside important aspects of burrow morphology at functionally relevant scales (microns to mm). Our focus is to determine whether commonly used alternative functional effect descriptors provide consistent information, and to investigate whether assessments of species contributions to benthic ecosystem process will benefit from the consideration of species behavior (bioirrigation) and/or various properties of associated biogenic structures.

## Results

**Figure 1:**
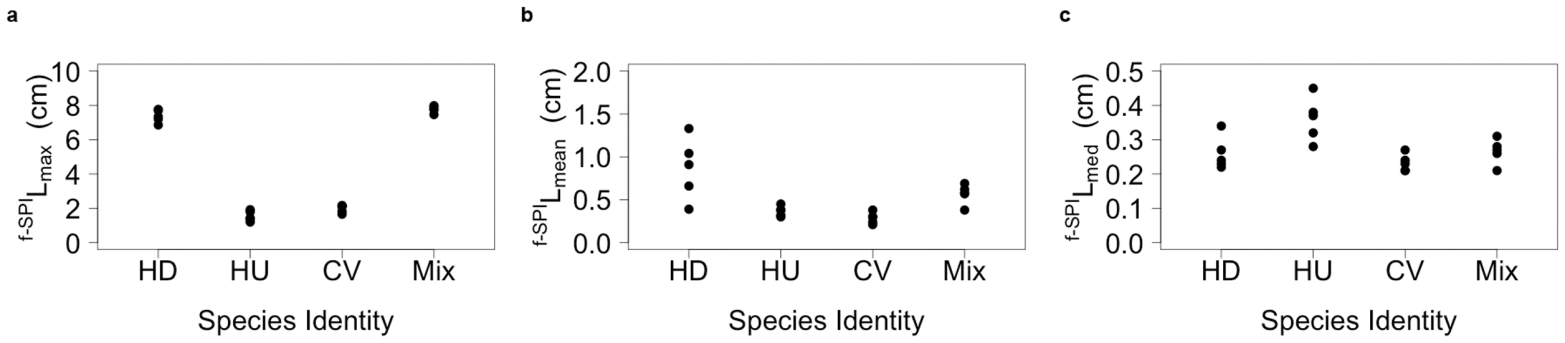
Sediment reworking associated with benthic invertebrates. Species-specific effects (n = 5) on the particle reworking metrics (a) ^f-SPI^L_max_, (b) ^f-SPI^L_mean_, and (c) ^f-SPI^L_med_. HD = *Hediste diversicolor*, HU = *Hydrobia ulvae*, CV = *Corophium volutator*, and Mix = species mixture comprising equal proportions (biomass ratio, 1:1:1) of HD, HU and CV.

The two-dimensional imaging of redistributed tracer particles (Supplementary Figures S2–S5) revealed little intra-specific variation but consistent inter-specific contrasts in redistribution profiles (Supplementary Figure S6). The rank order of species contributions, however, reflected subtle differences in the functional emphasis of each response variable (compare panels in Figure 1). The species contributing the greatest effect on particle redistribution were *Hediste diversicolor* for ^f-SPI^L_mean_, *Hydrobia ulvae* for ^f-SPI^L_med_, and the species mixture for ^f-SPI^L_max_. Overall, the maximum depth of vertical displacement (^f-SPI^L_max_, n = 5, ±95% CI; Figure 1a) ranged from 1.546 ± 0.374 cm for *Hydrobia ulvae* to 7.788 ± 0.249 cm for species in mixture, whilst the mean depth (^f-SPI^L_mean_, n = 5, ±95% CI; Figure 1b) ranged from 0.286 ± 0.081 cm for *Corophium volutator* to 0.866 ± 0.446 cm for *H. diversicolor* and the median depth (^f-SPI^L_med_, n = 5, ±95% CI; Figure 1c) ranged from 0.232 ± 0.031 cm for *C. volutator* to 0.360 ± 0.080 cm for *H. ulvae*. Closer examination of the minimum adequate models revealed distinct inter-specific differences in the depth of ^f-SPI^L_max_ (L-ratio = 68.592, d.f. = 3, p < 0.0001; Supplementary Model S3), ^f-SPI^L_mean_ (L-ratio = 19.182, d.f. = 3, p = 0.0003; Supplementary Model S1), and ^f-SPI^L_med_ (L-ratio = 11.505, d.f. = 3, p = 0.0093; Supplementary Model S2), but despite such subsurface mixing we were unable to determine any notable effect on surface boundary roughness (intercept only model, F = 0.3446, d.f. = 3, p = 0.7935; Supplementary Model S4). For ^f-SPI^L_max_, these inter-specific differences were distinct across all possible pairwise species permutations, with the effects of *C. volutator* (coefficient ± s.e. = −5.399 ± 0.195, t = −27.66, p < 0.0001) and *H. ulvae* (coefficient ± s.e. = −5.831 ± 0.215, t = −27.19, p < 0.0001) less than those of *H. diversicolor*, and the effects of the species mixture (coefficient ± s.e. = 0.411 ± 0.189, t = 2.17, p = 0.0454) greater than those of *H. diversicolor*. For ^f-SPI^L_mean_ (Figure 1b), the activities of *H. diversicolor* and the species mixture were indistinguishable from one another (coefficient ± s.e. = −0.298 ± 0.169, t = −1.766, p = 0.097) and resulted in deeper mean mixing levels than those of *C. volutator* and *H. ulvae*, which were also similar, albeit marginally, to one another (coefficient ± s.e. = 0.080 ± 0.039, t = 2.031, p = 0.059) but different to those of *H. diversicolor* and the species mixture (compare coefficients, Supplementary Model S1). The patterns of mixing shown by ^f-SPI^L_med_ (Figure 1c) reveal that the activities of *H. ulvae* (mean depth, n = 5, ±95% CI, = 0.360 ± 0.08 cm) were greater than those of *H. diversicolor* (coefficient ± s.e. = 0.100 ± 0.036, t = 2.774, p = 0.014), *C. volutator* (coefficient ± s.e. = 0.128 ± 0.031, t = 4.144, p < 0.001) and the species mixture (coefficient ± s.e. = 0.094 ± 0.033, t = 2.839, p = 0.012), which were all insignificant from one another (compare coefficients, Supplementary Model S2). Interestingly, in the case of ^f-SPI^L_med_, the degree of mixing in the species mixture did not necessarily reflect that of the most dominant species (*H. ulvae*) contribution in monoculture. Indeed, both *H. diversicolor* and *C. volutator* were clearly the most influential species across all three measures of particle redistribution (compare panels, Figure 1).

**Figure 2:**
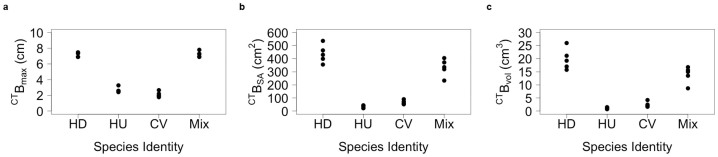
The extent of species-environment interaction associated with benthic invertebrates. Species-specific effects (n = 5) on burrow geometry metrics (a) ^CT^B_max_, (b) ^CT^B_SA_, and (c) ^CT^B_vol_. HD = *Hediste diversicolor*, HU = *Hydrobia ulvae*, CV = *Corophium volutator*, and Mix = species mixture comprising equal proportions (biomass ratio, 1:1:1) of HD, HU and CV.

Reconstruction of biogenic structures using computer tomography techniques revealed species-specific burrow systems that differed markedly in burrow density, structural morphology and depth distribution (Figure 2, Supplementary Figures S7–S9 and Supplementary Movie Sequences 1–4). *Hediste diversicolor* constructed extensive branched burrow galleries throughout the sediment profile (^CT^B_max_ range, 6.89–7.48 cm), *Corophium volutator* constructed multiple U-shape burrows that were confined to the surficial layers (^CT^B_max_ range, 1.77–2.66 cm), and *Hydrobia ulvae* constructed vertical I-shape excavations, albeit infrequently, to depths in excess of 2.4 cm (^CT^B_max_ range, 2.41–3.27 cm). These clear distinctions in species burrowing behavior led to concomitant changes in the maximum depth (^CT^B_max_, Figure 2a), surface area (^CT^B_SA_, Figure 2b) and lumen volume (^CT^B_vol_ Figure 2c) of the burrows but, when species were combined in mixture, the total was not necessarily additive (compare monoculture to mixture performance, Figure 2) and did not always match the maximum contributing species (comparison of mixture to *H. diversicolor* for: ^CT^B_max_, coefficient ± s.e. = 0.034 ± 0.213, t = 0.160, p = 0.875; ^CT^B_SA_, coefficient ± s.e. = −103.98 ± 42.17, t = −2.466, p = 0.025; ^CT^B_vol_, coefficient ± s.e. = −5.914 ± 2.283, t = −2.591, p = 0.020; Supplementary Models S5–S7). For ^CT^B_max_, the activities of *H. diversicolor* and the species mixture were indistinguishable from one another (coefficient ± s.e. = 0.034 ± 0.213, t = 0.160, p = 0.875) and were deeper than those of *H. ulvae* and they, in turn, were deeper than those of *C. volutator* (compare coefficients, Supplementary Model S5). ^CT^B_SA_ and ^CT^B_vol_ were, as expected, highly correlated (r = 0.99) and showed the same sequence of species performance (HD ≥ Mix > CV > HU; compare coefficients, Supplementary Models S6–S7). ^CT^B_SA_ and ^CT^B_vol_ in species mixture were intermediate relative to performance observed in respective monocultures (Figure 2).

**Figure 3:**
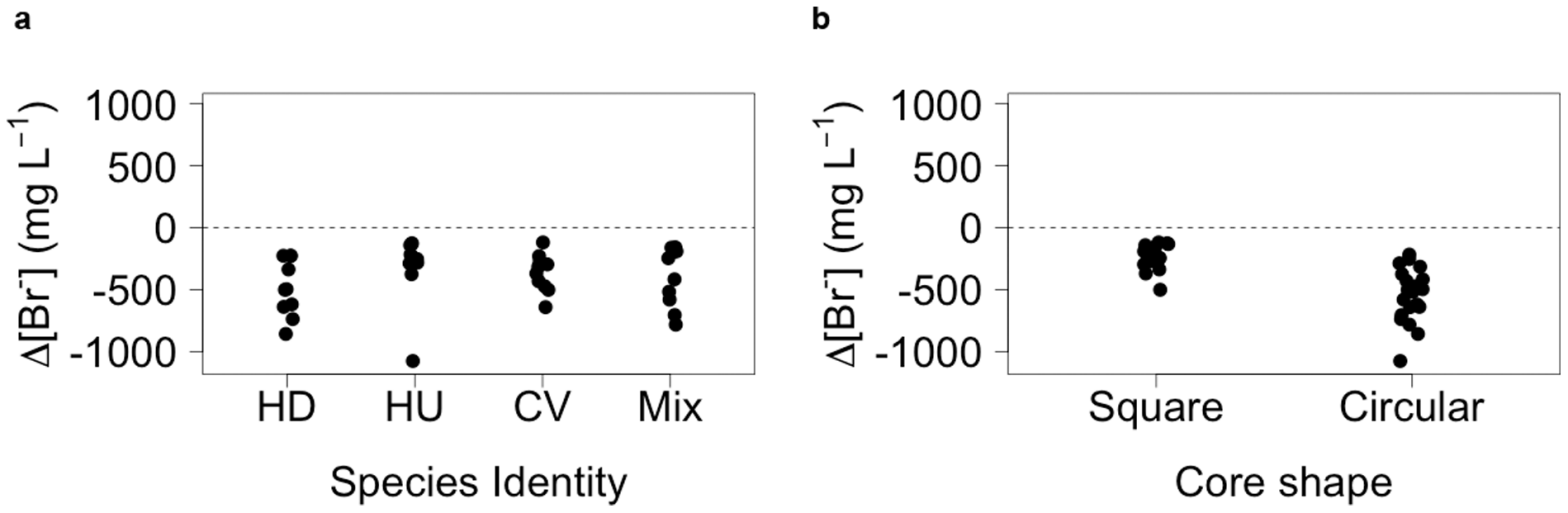
Independent effects of (a) species identity and (b) core shape on bioirrigation activity (Δ[Br^−^], mg L^−1^). Negative values indicate increased bioirrigation activity. For clarity, jitter (a = 0.05) has been applied to the x = argument of the plot function to avoid over-plotting. HD = *Hediste diversicolor*, HU = *Hydrobia ulvae*, CV = *Corophium volutator*, and Mix = species mixture comprising equal proportions (biomass ratio, 1:1:1) of HD, HU and CV.

There were independent effects of species identity and core shape on bioirrigation behavior (Figure 3). For species, most bioirrigation activity (mean Δ[Br^−^], n = 10, ±95% CI = −486.79 ± 162.92 mg L^−1^) was performed by *H. diversicolor*, followed by the remaining species treatments (coefficient ± s.e.: CV, 103.37 ± 53.16, t = 1.944, p = 0.060; Mix, 126.96 ± 45.27, t = 2.805, p = 0.008; HU, 139.89 ± 53.03, t = 2.638, p = 0.012; Figure 3a), which were generally indistinguishable from one another (compare coefficients, Supplementary Model S8). In general, irrespective of species identity, there was a greater amount of bioirrigation (mean Δ[Br^−^], n = 20, ±95% CI) in the circular cores (circular, −546.15 ± 102.11 mg L^−1^; square, −234.11 ± 43.79 mg L^−1^) relative to the square cores (coefficient ± s.e. = −325.20 ± 41.88, t = −7.765, p < 0.0001; Figure 3b).

**Figure 4:**
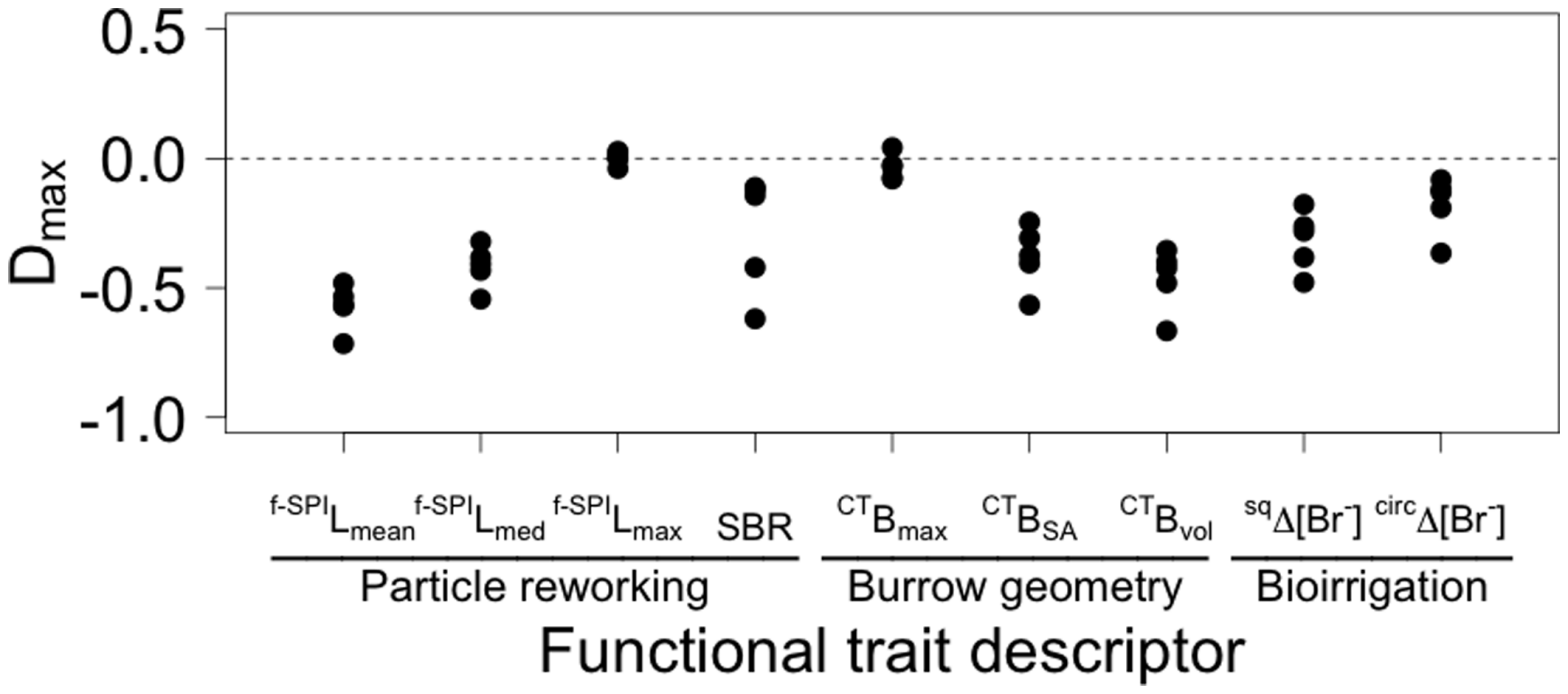
Observed yield (D_max_) of different components of particle reworking, burrow geometry and bioirrigation behaviour for species mixtures relative to species monocultures with equivalent biomass. The observed yield indicates whether a species mixture outperforms (overyielding, D_max_ > 0) or underperforms (underyielding, D_max_ < 0) relative to the maximally performing species in monoculture, and provides an indication of how species interactions may influence species contributions to ecosystem process.

For the components of particle redistribution, properties of biogenic structure and bioirrigation behavior that were measured, we found no evidence of overyielding (Figure 4). With the exception of ^f-SPI^L_max_ and ^CT^B_max_, which maintain the equivalent level of process to *H. diversicolor* in monoculture, all other response variables exhibit a change in the mean (±95% CI, n = 5) level of process when in species mixture (ranging from −0.177 ± 0.139 for ^circ^*Δ*[Br] to −0.573 ± 0.108 for ^f-SPI^L_mean_; Figure 4).

## Discussion

We have demonstrated distinct species-specific differences in the redistribution of sediment particles, fluid transport, and the relative geometric properties of biogenic structures, three major mechanisms by which species mediate important ecosystem functions in the marine benthos^15^. Our data show, however, that evaluations of the contributions that species make to each of these categories can be misleading when based on a single, or a limited set of, functional effect descriptors. We find that different properties of routinely used data on the vertical redistribution of sediment particles reflect either short-term infaunal activity in the uppermost region of the sediment profile (^f-SPI^L_med_), less frequently occurring infaunal activity at depth (^f-SPI^L_max_), or the relative balance between the minimum and maximum depth of mixing (^f-SPI^L_mean_). Similarly, bioirrigation (Δ[Br^−^]) poorly related to burrow geometry, suggesting that the propensity to irrigate burrows was more important than the dimensions of burrow structures^35^. Surface boundary roughness was unable to discriminate between species, despite contrasts in the lifestyle and residency depth of the species under study, as all species were capable of redistributing surficial sediment. Importantly, these differences in emphasis across closely related functional effect descriptors lead to contradictory interpretations of the functional standing of a species because each descriptor integrates aspects of species behavior that are not universally attributable. Moreover, it is clear that routinely used proxies for various ecosystem processes only provide partial coverage of the mechanisms that contribute to functioning. Our analyses show, however, that these interpretative inconsistencies can be reconciled, and poor levels of discriminatory power can be improved, when elements of species behavior and/or properties of biogenic structures are considered in concert with other ‘biological’ derived traits because the former are critical in determining the functional expression of traits^11^,^14^.

Here, we used three-dimensional reconstructions of burrow geometry (^CT^B_SA_, ^CT^B_vol_, ^CT^B_max_) and two dimensional assessments of surface boundary roughness and bioirrigation activity (Δ[Br^−^]) to gain important insights about the relative importance of the interaction between species behavior and biogenic structures. We found that their were distinct inter-specific differences in species behavior and burrow morphology (*H. diversicolor*, extensive inter-weaving galleries extending to ~7.5 cm depth with multiple openings at the sediment-water interface; *C. volutator*, U-shaped burrows extending to ~2.6 cm depth; *H. ulvae*, I- and J-shaped vertical shafts to ~3.3 cm) that dictate whether fluid transport is primarily diffusive or advective^36^. Our methodology did not allow us to determine the temporal pattern of bioirrigation activity, but we were able to identify distinct species-specific differences in relative performance. Whilst some of this information is known, at least qualitatively, it is important to emphasize that our approach of using multiple functional effect descriptors was instrumental in providing novel functionally relevant information. For instance, the behavior^37^,^38^ and effects of *H. ulvae* on ecosystem process and functioning have been extensively described^5^,^33^,^39^,^40^, but the spatial distribution, morphology and significance of their burrow system has not previously been recognized or quantified. Prior summaries of the functional role of *H. ulvae* have emphasized surficial sediment destabilisation through surface browsing and disruption^41^, but the data presented here suggests that *H. ulvae* significantly contributes to much deeper sediment destabilisation, especially in areas of high sediment moisture content^38^ or patchy resource supply^5^ where activity levels are elevated. Such information provides mechanistic explanations for otherwise inconsistent observations of species contributions to ecosystem functioning, and reinforces the view that that the functional role of a species is not static and cannot be adequately summarized without reference to variation in species behavior and use of multiple functional parameters^42^.

Whilst we were able to identify inter-specific differences in functional effects across our monocultures, our results also highlight the importance of considering whether the functional role of species changes when in the presence of other species. We found that particle reworking, bioirrigation, burrow surface area and burrow volume all fell below expected values when species were in mixture (underyielding, D_max_ < 0), an outcome consistent with findings elsewhere^1^,^43^ that highlights the importance of the modifying effects (positive and negative) of species interaction^1^. We interpret these effects to be in relation to competition for space and/or resources^44^,^45^, rather than being due to density effects^46^,^47^, lending support to the view that the functional role of a species may reflect a behavioral response to the biotic setting rather than the presence or absence of particular biological traits^48^; the exploratory and prospecting behavior of *H. diversicolor*, for example, is known to disrupt *C. volutator* burrows, resulting in the relocation of individuals and additional burrow construction activity^5^,^17^,^39^ that alters the relative amount of time allocated to other functionally important behaviors, such as bioirrigation^49^. Such context-dependent behavioural responses constitute activity that is additional to, and distinct from, activity associated with routine behavior^17^ and may fundamentally change the functional role of a species over extended timescales. An immediate challenge, therefore, is to distinguish the functional effect of transient changes in behavior^5^,^18^,^25^,^26^,^27^ from those associated with acclimation or adaptation to novel abiotic and biotic circumstances^50^. This matters because the structural properties of a community may well be conserved after a perturbation, or following a period of directional forcing, but the functional role of individual species may fundamentally change relative to the pre-disturbance standard^50^,^51^. Failure to collate this information dramatically reduces the accuracy and predictive capabilities of next generation ecosystem models but, as the effects of core shape reveal in the present study, it will also be important to ensure that efforts towards this goal standardize experimental protocols and account for differences in experimental setting^52^,^53^. Nevertheless, a substantial revision of previously described functional effect groupings is required before the true value of biodiversity for humanity can be ascertained.

## Methods

Surficial sediment (less than 3 cm depth; mean particle size, 54.80 μm; mud content, 55.93%) and three co-occurring functionally contrasting inter-tidal invertebrates (the polychaete *Hediste diversicolor*, the gastropod *Hydrobia ulvae* and mud shrimp *Corophium volutator*) were collected from the mid-shore at Breydon water, Great Yarmouth, UK (N52° 37.030′, E01° 41.390′) and returned to the *Biodiversity and Ecosystem Futures Facility* at the University of Southampton to acclimatise to laboratory conditions (5 days). Sediment was sieved (500 μm mesh) in a seawater (sand filtered, UV sterilized and salinity 33) bath to remove macrofauna, allowed to settle for 48 h to retain the fine fraction (less than 63 μm) and homogenized. Each aquarium was filled to a depth of 8 cm with sediment homogenate overlain by 4 cm seawater. Fauna were not added until the lower regions of the cores showed evidence of reducing conditions (visible anoxic microniche formation). Overlying seawater was replaced after 24 h to remove excess nutrients associated with assembly. Aquaria were maintained at 12 ± 0.1°C under a 12:12 h light (Aqualine T5 Reef White 10 K fluorescent light tubes, Aqua Medic) cycle and were continually aerated. Although inter-tidal species exhibit a rhythm of activity associated with the tidal cycle, it was not necessary to incorporate an immersion-emersion cycle into our experimental design because the biological rhythms in the species under study are known to be endogenous, i.e. they persist long after an organism is isolated from periodic tidal cues that alter their behavior.

Replicate (n = 5) invertebrate communities were assembled in monoculture (*Hediste diversicolor*, HD; *Hydrobia ulvae*, HU; or *Corophium volutator*, CV) and in a mixture (Mix) of all three species (biomass fixed at 1 g wet weight aquaria^−1^; ~127 g m^−2^, equivalent to a monoculture density of ~850, ~22,000 or ~25,000 ind. m^−2^ for HD, HU and CV respectively) in each of two types of aquaria (square vs circular) that shared an identical volume of habitat (1178 cm^3^). Square aquaria (internal dimensions 8.86 × 8.86 cm × 15.0 cm, n = 20) were required to facilitate quantification of particle reworking using 2-dimensional photographic imaging, whilst circular aquaria (internal diameter = 10 cm, 15 cm tall, n = 20) were required to facilitate rotational quantification of biogenic structures using 3-dimensional computer tomography (CT) imaging.

Faunal mediated sediment particle reworking in the square aquaria was estimated non-invasively using a sediment profile imaging camera (Canon 400D set to 10 s exposure, aperture f5.6 and ISO 400; 3888 × 2592 pixels, i.e. 10.1 megapixels, effective resolution of sediment 56 × 56 μm per pixel), optically modified to allow preferential imaging of fluorescently labeled sand-based particulate tracers under UV light (f-SPI^54^). These tracers were evenly distributed over the entire sediment surface (~3 mm depth) 24 h after faunal addition to ensure that elevated levels of activity associated with initial burrow construction were not incorporated into estimates of infaunal mixing. The redistribution of luminophores (15 g aquarium^−1^, pink colour, size class less than 125 μm, mean particle size ~ 80 μm; Brian Clegg Ltd., UK) was determined from stitched composite images (RGB colour, JPEG compression) of all four sides of each aquarium obtained in a UV illuminated imaging box^55^ after 6 days using a custom-made semi-automated macro that runs within *ImageJ* (Version 1.47), a java-based public domain program developed at the US National Institutes of Health (http://rsb.info.nih.gov/ij/index.html, Rasband, W., ImageJ., (1997), Date of access 10/07/2013). From these data, the median (^f-SPI^L_med_, typical short-term depth of mixing), maximum (^f-SPI^L_max_, maximum extent of mixing over the long-term) and mean (^f-SPI^L_mean_, time dependent indication of mixing) mixed depth of particle redistribution were calculated. In addition, the maximum vertical deviation of the sediment-water interface (upper – lower limit = surface boundary roughness, SBR) provided an indication of surficial activity.

Quantification of biogenic structures in the circular aquaria was achieved using a 225/450 kV Nikon/Metris custom designed micro-focus computer tomography scanner housed within the μ-VIS imaging centre, University of Southampton. Batches of 5 aquaria were stacked and secured in a custom-made holding brace to ensure stability during scanning. During each acquisition, the aquaria were rotated through 360° whilst collecting 3142 projections averaging over 8 frames per 250 ms projection. X-ray conditions were set to 300 kV and 326 μA with a 3 mm Cu filter, and an XRD 1621 CN3 H5 PerkinElmer flat panel detector was used to collect the images. In the resulting images, levels of grey scale reflect the level of x-ray attenuation caused by variation in bulk density^56^. Hence, brighter pixels represent denser material (sediment) and darker pixels represent less dense material (burrow voids). Raw image slices (n = 2000 aquarium^−1^, voxel resolution = 81 μm) were processed as follows: First, the projection data was reconstructed using *CTPro3D* (v. XT 2.2 service pack 10, Nikon Metrology, UK) and *CTAgent* (v. XT 2.2 service pack 10, Nikon Metrology, UK). The reconstructed volumes were converted to 8 bit format using FIJI^57^ in order to reduce file sizes and computational loading. Finally, these images were opened as a 3D project in *VGStudio* (v. 2.1 Volume Graphics GmbH, Germany). From these data, regions of interest were segmented using a threshold based seed point growing algorithm from which the surface area (^CT^B_SA_, an important determinant of microbial-mediated biogeochemical cycling), volume (^CT^B_vol_, an indication of the extent of bioengineering and bioirrigation) and maximum depth of any biogenic features (^CT^B_max_) were calculated.

Bioirrigation was estimated in both square and circular aquaria from absolute changes in the concentration of the inert tracer sodium bromide (Δ[Br^−^], mg l^−1^; negative values indicate increased bioirrigation activity^58^) over an 8 h period (daytime, in square and circular cores) on day 6, determined using a Tecator flow injection auto-analyser (FIA Star 5010 series).

In order to assess whether there were any effects of species interactions on our dependent variables, we compared performance in monoculture to performance in mixture^59^. Overyielding (D_max_ > 0) occurs when a mixture outperforms expectations based on the corresponding monocultures.

Linear regression models were developed for the dependent variables (^f-SPI^L_med_, ^f-SPI^L_max_, ^f-SPI^L_mean_, SBR, ^CT^B_SA_, ^CT^B_vol_, ^CT^B_max_, Δ[Br^−^]) with the independent nominal variable species identity (SPID) or, for bioirrigation, the nominal variables SPID and core shape (square versus circular). In all cases, we found evidence for a violation of homogeneity of variance so a *VarIdent* variance-covariate structure and a generalised least squares (GLS) estimation procedure^60^ that allows the residual spread to vary with individual explanatory variables was applied. For all of the bioturbation analyses, the residual spread varied with species identity whilst, for the bioirrigation analysis, the residual spread varied with both core shape and species identity. We determined the optimal fixed-effects structure for each model using backward selection informed by Akaike Information Criteria (AIC) and inspection of model residual patterns. The optimal variance covariate structure was determined by comparing the initial ANOVA model without variance structure to the equivalent GLS model incorporating specific variance structures using AIC and visualisation of model residuals obtained by restricted maximum likelihood (REML) estimation. The optimal fixed structure was then determined by applying backward selection using the likelihood ratio test obtained using maximum likelihood (ML) estimation. All analyses were performed in R (v. 2.15.2) using the *nlme* package^61^. All minimal adequate models and their associated coefficient tables are presented in supplementary material.

## Supplementary Material

Supplementary Information Supplementary Information

Supplementary Information Supplementary movie 1

Supplementary Information Supplementary movie 2

Supplementary Information Supplementary movie 3

Supplementary Information Supplementary movie 4
