# Supplementary material for: Characterizations of how species mediate ecosystem properties require more comprehensive functional effect descriptors
Source: Sci Rep. 2014 Sep 24;4:6463. doi: 10.1038/srep06463 (PMC5377471; doi:10.1038/srep06463)
Supplement: Supplementary Information [file srep06463-s1.pdf]

**Supplementary Material**

# Characterizations of how species mediate ecosystem properties require more comprehensive functional effect descriptors

R. Hale[1], M.N. Mavrogordato[2], T.J. Tolhurst[1] & M. Solan[3].

[1]University of East Anglia, School of Environmental Science, Norwich, NR4 7TJ, UK, [2]University of Southampton, Engineering and the Environment, Highfield, Southampton, SO17 1BJ, UK, [3]Ocean and Earth Science, National Oceanography Centre, Southampton, University of Southampton, Waterfront Campus, European Way, Southampton, SO14 3ZH, UK.

## Statistical model summary

Summary of the statistical analyses for our 8 statistical models (Models S1 to S8). For each model, we list the initial linear regression model, the minimal adequate model with GLS estimation, and a summary of the coefficient table. The coefficients indicate the relative performance of each treatment level relative to the relevelled baseline (as indicated). Coefficients ± SE and t-values are presented alongside corresponding significance values (in parentheses). Abbreviations: HD, *Hediste diversicolor*; HU, *Hydrobia ulvae*; CV, *Corophium volutator*; Mix, 1:1:1 mix of HD, HU and CV; SPID, Species identity [HD, HU, CV, Mix]; CS, core shape [square, sq or circular, circ].

**Supplementary Model S1|** Mean maximum mixed depth of particle reworking ($^{f\text{-}SPI}L_{mean}$, cm)

Initial linear regression model:

lm($^{f\text{-}SPI}L_{mean}$ ~ as.factor(SPID))

Minimal adequate model:

gls($^{f\text{-}SPI}L_{mean}$ ~ as.factor(SPID),

weights = varIdent(form = ~1|as.factor(SPID)), method = 'REML')

Coefficient Table

Intercept ± SE (when baseline is for HD): 0.866 ± 0.1607047, t = 5.388766, p = 0.0001.

| | HD | HU | CV | Mix |
|---|---|---|---|---|
| HD | - | -0.500 ± 0.1628558<br>-3.070201<br>(**0.0073**) | -0.580 ± 0.163346<br>-3.550739<br>(**0.0027**) | -0.298 ± 0.168760<br>-1.765819<br>(0.0965) |
| HU | 0.500 ± 0.162856<br>3.070201<br>(**0.0073**) | - | -0.080 ± 0.039395<br>-2.030692<br>(0.0592) | 0.202 ± 0.057879<br>3.490028<br>(**0.0030**) |
| CV | 0.580 ± 0.163346<br>3.550739<br>(**0.0027**) | 0.080 ± 0.039395<br>2.030692<br>(0.0592) | - | 0.282 ± 0.059245<br>4.759875<br>(**0.0002**) |
| Mix | 0.298 ± 0.168760<br>1.765819<br>(0.0965) | -0.202 ± 0.057879<br>-3.490028<br>(**0.0030**) | -0.282 ± 0.059245<br>-4.759875<br>(**0.0002**) | - |

**Supplementary Model S2 |** Median maximum mixed depth of particle reworking ($^{f\text{-}SPI}L_{med}$, cm)

Initial linear regression model:

lm($^{f\text{-}SPI}L_{med}$ ~ as.factor(SPID))

Minimal adequate model:

gls($^{f\text{-}SPI}L_{med}$ ~ as.factor(SPID), weights = varIdent(form = ~1|as.factor(SPID)), method = 'REML')

Coefficient Table

Intercept ± SE (when baseline is for HD): 0.260 ± 0.02167948, t = 11.992906, p = <0.0001.

| | HD | HU | CV | Mix |
|---|---|---|---|---|
| HD | - | 0.100 ± 0.036056<br>2.773501<br>(**0.0136**) | -0.028 ± 0.024372<br>-1.148854<br>(0.2675) | 0.006 ± 0.027129<br>0.221163<br>(0.8278) |
| HU | -0.100 ± 0.036056<br>-2.773501<br>(**0.0136**) | - | -0.128 ± 0.030887<br>-4.144153<br>(**0.0008**) | -0.094 ± 0.033106<br>-2.839374<br>(**0.0118**) |
| CV | 0.028 ± 0.024372<br>1.148854<br>(0.2675) | 0.128 ± 0.030887<br>4.144153<br>(**0.0008**) | - | 0.034 ± 0.019748<br>1.721657<br>(0.1044) |
| Mix | -0.006 ± 0.027129<br>-0.221163<br>(0.8278) | 0.094 ± 0.033106<br>2.839374<br>(**0.0118**) | -0.034 ± 0.019748<br>-1.721657<br>(0.1044) | - |

**Supplementary Model S3 |** Maximum mixed depth of particle reworking ($^{f\text{-}SPI}L_{max}$, cm)

Initial linear regression model:

lm($^{f\text{-}SPI}L_{max}$ ~ as.factor(SPID))

Minimal adequate model:

gls($^{f\text{-}SPI}L_{max}$ ~ as.factor(SPID), weights = varIdent(form = ~1|as.factor(SPID)), method = 'REML')

Coefficient Table

Intercept ± SE (when baseline is for HD): 7.37668 ± 0.1667988, t = 44.22501, p = <0.0001.

| | HD | HU | CV | Mix |
|---|---|---|---|---|
| HD | - | -5.831 ± 0.214456<br>-27.19044<br>(**<0.0001**) | -5.399 ± 0.195204<br>-27.65770<br>(**<0.0001**) | 0.411 ± 0.189453<br>2.16993<br>(**0.0454**) |
| HU | 5.831 ± 0.214456<br>27.19044<br>(**<0.0001**) | - | 0.43224 ± 0.168678<br>2.56252<br>(**0.0209**) | 6.24224 ± 0.161988<br>38.53526<br>(**<0.0001**) |
| CV | 5.3989 ± 0.195204<br>27.65770<br>(**<0.0001**) | -0.4322 ± 0.168678<br>-2.56252<br>(**0.0209**) | - | 5.81000 ± 0.135475<br>42.88607<br>(**<0.0001**) |
| Mix | -0.4111 ± 0.189453<br>-2.16993<br>(**0.0454**) | -6.242 ± 0.161988<br>-38.53526<br>(**<0.0001**) | -5.810 ± 0.135475<br>-42.88607<br>(**<0.0001**) | - |

**Supplementary Model S4 |** Surface boundary roughness (SBR, cm)

Initial linear regression model:

lm(SBR ~ as.factor(SPID))

No minimal adequate model, intercept only (SPID, F = 0.3446, d.f. = 3, p = 0.7935).

**Supplementary Figure S1 |** Summary of Surface Boundary Roughness (SBR, cm) observed for all species treatments. Data is shown for information only, as we found no effect of species identity.

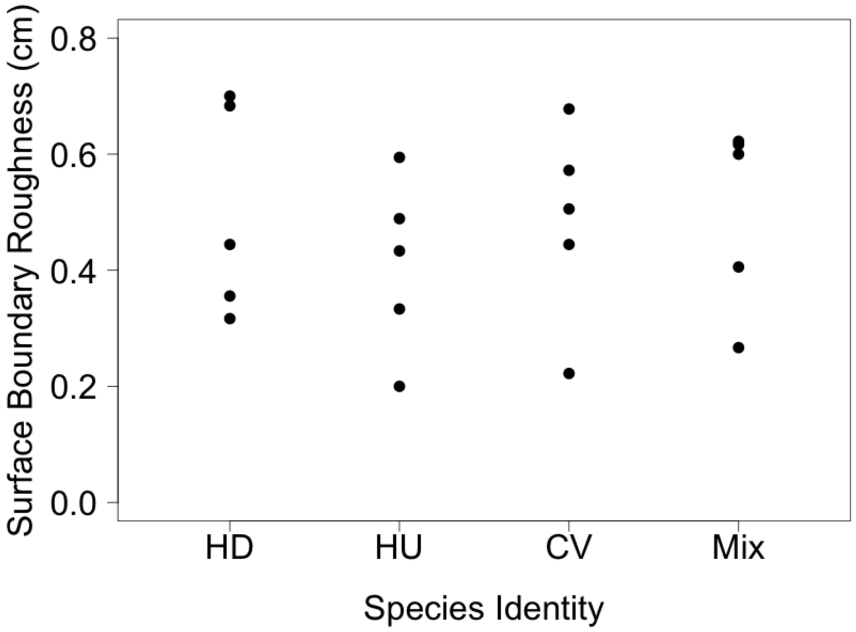

**Supplementary Model S5 |** Maximum burrow depth ($^{CT}B_{max}$, cm)

Initial linear regression model:

lm($^{CT}B_{max}$ ~ as.factor(SPID))

Minimal adequate model:

lm($^{CT}B_{max}$ ~ as.factor(SPID))

Coefficient Table

Intercept ± SE (when baseline is for HD): 7.2020 ± 0.1505, t = 47.85, p = <0.0001.

| | HD | HU | CV | Mix |
|---|---|---|---|---|
| HD | - | -4.5380 ± 0.2129<br>-21.318<br>(**<0.0001**) | -5.0940 ± 0.2129<br>-23.930<br>(**<0.0001**) | 0.0340 ± 0.2129<br>0.16<br>(0.0875) |
| HU | 4.5380 ± 0.2129<br>21.318<br>(**<0.0001**) | - | -0.5560 ± 0.2129<br>-2.612<br>(**0.0189**) | 4.5720 ± 0.2129<br>21.478<br>(**<0.0001**) |
| CV | 5.0940 ± 0.2129<br>23.930<br>(**<0.0001**) | 0.5560 ± 0.2129<br>2.612<br>(**0.0189**) | - | 5.1280 ± 0.2129<br>24.089<br>(**<0.0001**) |
| Mix | -0.0340 ± 0.2129<br>-0.16<br>(0.0875) | -4.5720 ± 0.2129<br>-21.478<br>(**<0.0001**) | -5.1280 ± 0.2129<br>-24.089<br>(**<0.0001**) | - |

**Supplementary Model S6 |** Burrow surface area ($^{CT}B_{SA}$, $cm^2$)

Initial linear regression model:

lm($^{CT}B_{SA}$ ~ as.factor(SPID))

Minimal adequate model:

gls($^{CT}B_{SA}$ ~ as.factor(SPID), weights = varIdent(form = ~1|as.factor(SPID)), method = 'REML')

Coefficient Table

Intercept ± SE (when baseline is for HD): 436.9088 ± 30.58431, t = 14.285389, p = <0.0001.

| | HD | HU | CV | Mix |
|---|---|---|---|---|
| HD | - | -403.743 ± 30.93032<br>-13.053297<br>(**<0.0001**) | -370.351 ± 31.37015<br>-11.805855<br>(**<0.0001**) | -103.9783 ± 42.1721<br>-2.465569<br>(**0.0254**) |
| HU | 403.7427 ± 30.93032<br>13.053297<br>(**<0.0001**) | - | 33.3913 ± 8.364851<br>3.991856<br>(**0.0010**) | 299.764 ± 29.40022<br>10.195991<br>(**<0.0001**) |
| CV | 370.3514 ± 31.37015<br>11.805855<br>(**<0.0001**) | -33.3913 ± 8.364851<br>-3.991856<br>(**0.0010**) | - | 266.373 ± 29.86259<br>8.919960<br>(**<0.0001**) |
| Mix | 103.9783 ± 42.17213<br>2.465569<br>(**0.0254**) | -299.764 ± 29.40022<br>-10.195991<br>(**<0.0001**) | -266.373 ± 29.86259<br>-8.919960<br>(**<0.0001**) | - |

**Supplementary Model S7 |** Burrow volume ($^{CT}B_{vol}$, $cm^3$)

Initial linear regression model:

lm($^{CT}B_{vol}$ ~ as.factor(SPID))

Minimal adequate model:

gls($^{CT}B_{vol}$ ~ as.factor(SPID), weights = varIdent(form = ~1|as.factor(SPID)), method = 'REML')

Coefficient Table

Intercept ± SE (when baseline is for HD): 19.829278 ± 1.796173, t = 11.039737, p = <0.0001.

| | HD | HU | CV | Mix |
|---|---|---|---|---|
| HD | - | -18.7110 ± 1.08029<br>-10.378262<br>(**<0.0001**) | -17.4040 ± 1.85599<br>-9.377166<br>(**<0.0001**) | -2.5905 ± 2.28300<br>-2.590540<br>(**0.0197**) |
| HU | 18.7110 ± 1.08029<br>10.378262<br>(**<0.0001**) | - | 1.3071 ± 0.49264<br>2.653182<br>(**0.0174**) | 12.7968 ± 1.41777<br>9.025995<br>(**<0.0001**) |
| CV | 17.4040 ± 1.85599<br>9.377166<br>(**<0.0001**) | -1.3071 ± 0.49264<br>-2.653182<br>(**0.0174**) | - | 11.4897 ± 1.48470<br>7.738784<br>(**<0.0001**) |
| Mix | 2.5905 ± 2.28300<br>2.590540<br>(**0.0197**) | -12.7968 ± 1.41777<br>-9.025995<br>(**<0.0001**) | -11.4897 ± 1.48470<br>-7.738784<br>(**<0.0001**) | - |

**Supplementary Model S8 |** Bioirrigation ($\Delta[Br^-]$, mg $L^{-1}$)

Initial linear regression model:

lm($\Delta[Br^-]$ ~ as.factor(SPID)* as.factor(CS))

Minimal adequate model:

gls($\Delta[Br^-]$ ~ as.factor(SPID) + as.factor(CS),

weights = varIdent(form = ~ 1|as.factor(SPID) * as.factor(CS)), method = 'REML')

Coefficient tables over page - >

Coefficient Tables

Intercept ± SE (when baseline is for HD in square cores): -321.8755 ± 43.33559, t = -7.427508, p = <0.0001. Core shape (CS) is denoted by subscripted text (circ =circular, sq = square). Note that, for bioirrigation, increasingly negative coefficient values indicate greater species contributions.

| | HD | HU | CV | Mix |
|---|---|---|---|---|
| HD | - | 139.8912 ± 53.03241<br>2.637844<br>**(0.0124)** | 103.3679 ± 53.16061<br>-1.944446<br>(0.0599) | 126.9681 ± 45.26761<br>2.804694<br>**(0.0082)** |
| HU | -139.8912 ± 53.03241<br>-2.637844<br>**(0.0124)** | - | -36.5233 ± 49.41169<br>-0.739163<br>(0.4647) | -12.9294 ± 35.25443<br>-0.366746<br>(0.7160) |
| CV | -103.3679 ± 53.16061<br>-1.944446<br>(0.0599) | 36.5233 ± 49.41169<br>0.739163<br>(0.4647) | - | 23.5939 ± 40.97514<br>0.575810<br>(0.5684) |
| Mix | -126.9618 ± 45.26761<br>-2.804694<br>(**0.0082**) | 12.9294 ± 35.25443<br>0.366746<br>(0.7160) | -23.5939 ± 40.97514<br>-0.575810<br>(0.5684) | - |

CS coefficient table over page ->

| | $CS_{sq}$ | $CS_{circ}$ |
|---|---|---|
| $CS_{sq}$ | - | -325.1956 ± 41.87742<br>-7.765417<br>**(<0.0001)** |
| $CS_{circ}$ | 325.1956 ± 41.87742<br>7.765417<br>**(<0.0001)** | - |

## Particle Reworking

Particle reworking was determined using fluorescent sediment profile imaging (f-SPI, see Methods in main manuscript) and an optically distinct particulate tracer (luminophores, shown in red). The raw images (four aquarium sides are stitched together) used to determine the sediment particle reworking profiles are presented. The scale for all images is indicated in the lower panel of each figure.

**Supplementary Figure S2 |** Replicate (n=5) f-SPI images for *Hediste diversicolor*.

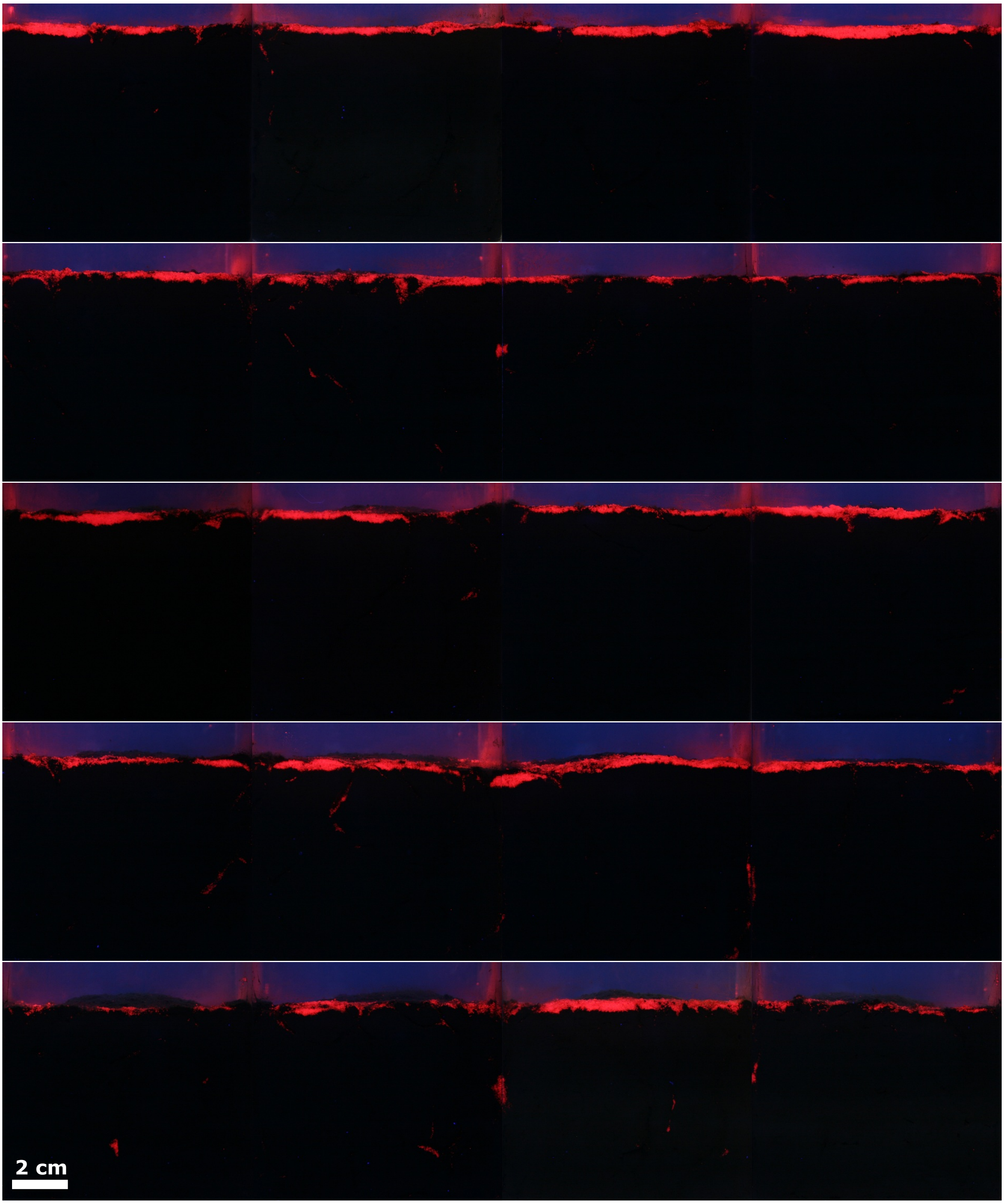

**Supplementary Figure S3 |** Replicate (n=5) f-SPI images for *Hydrobia ulvae*.

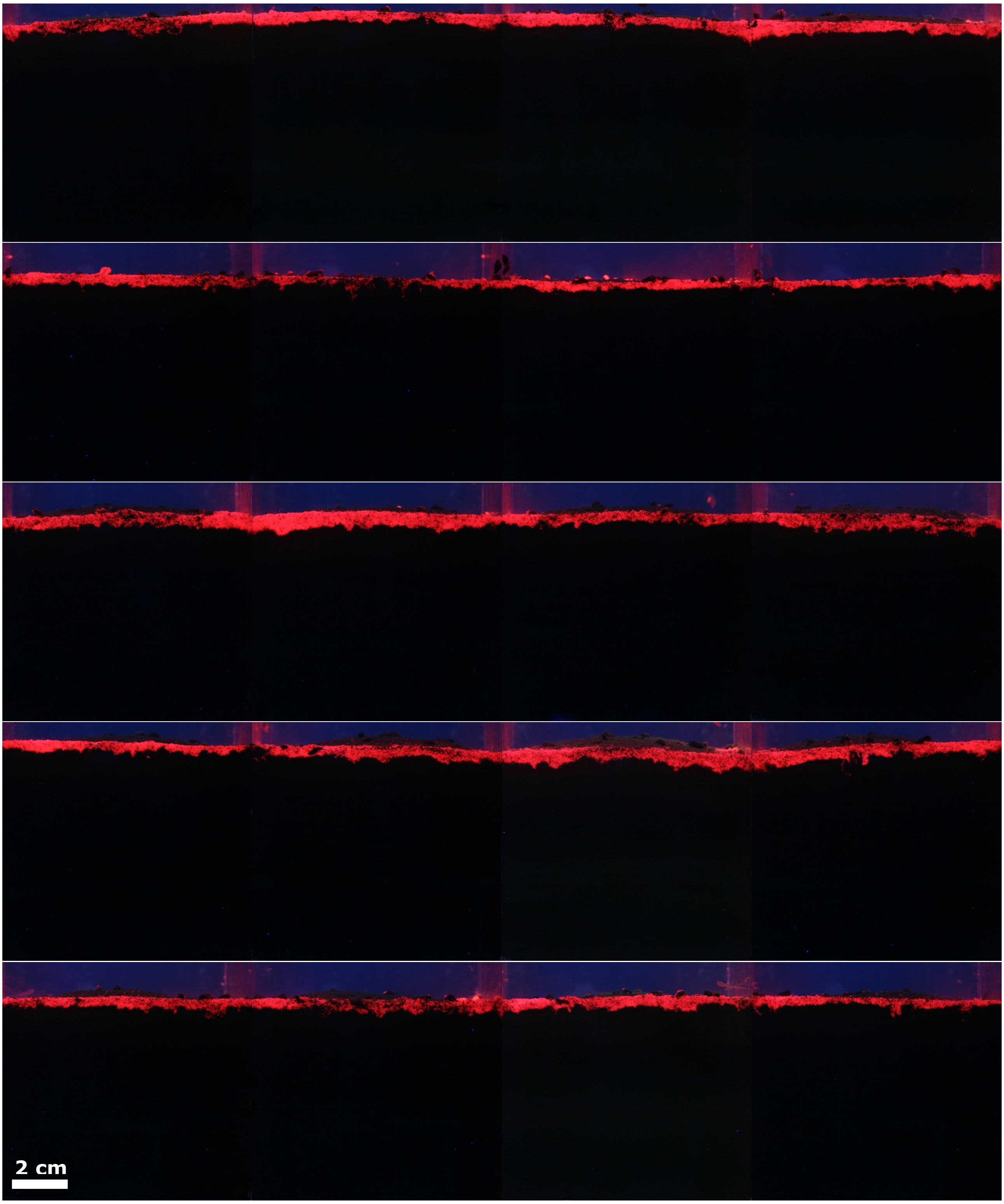

**Supplementary Figure S4 |** Replicate (n=5) f-SPI images for *Corophium volutator*.

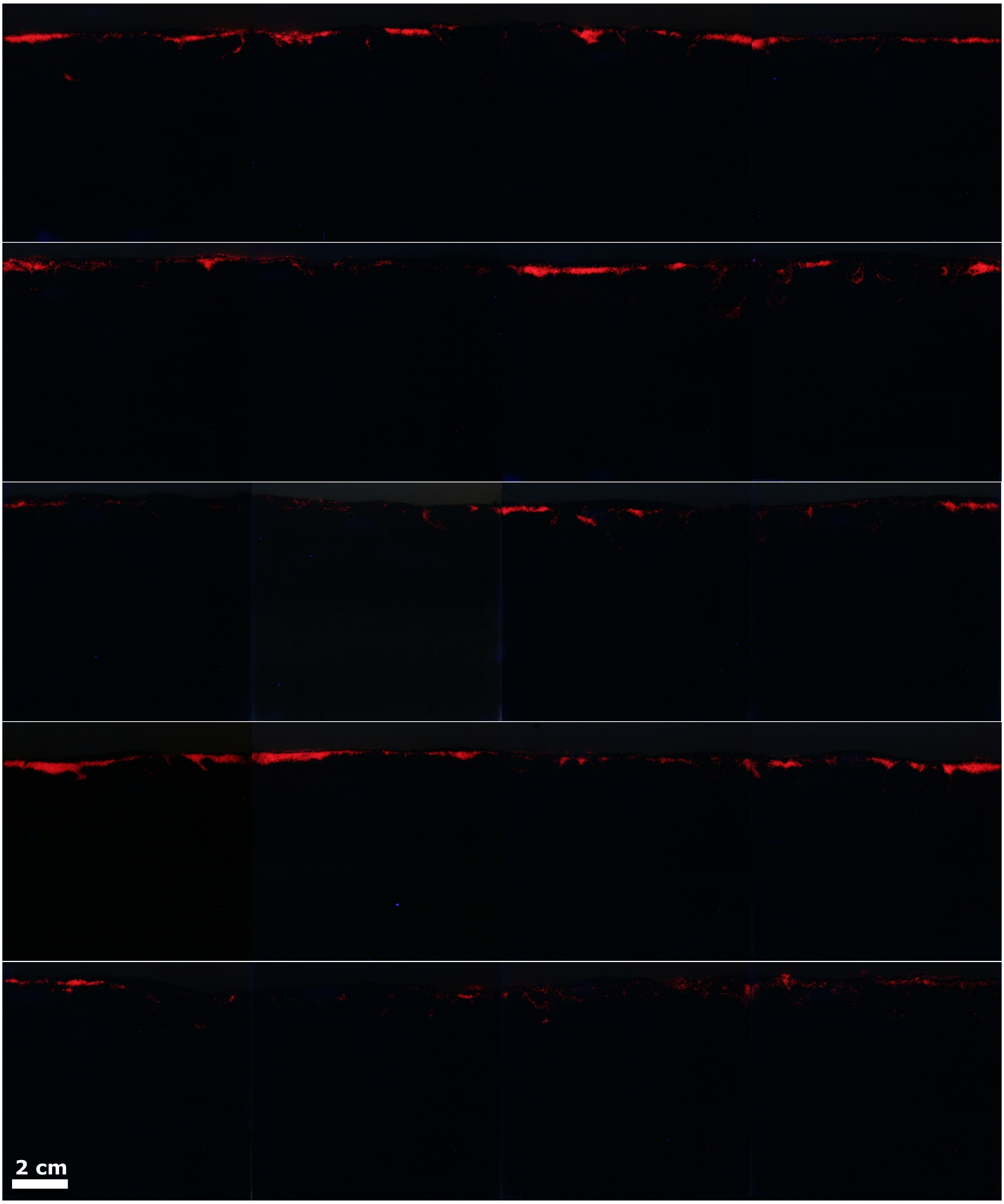

**Supplementary Figure S5 |** Replicate (n=5) f-SPI images for the mixed treatment.

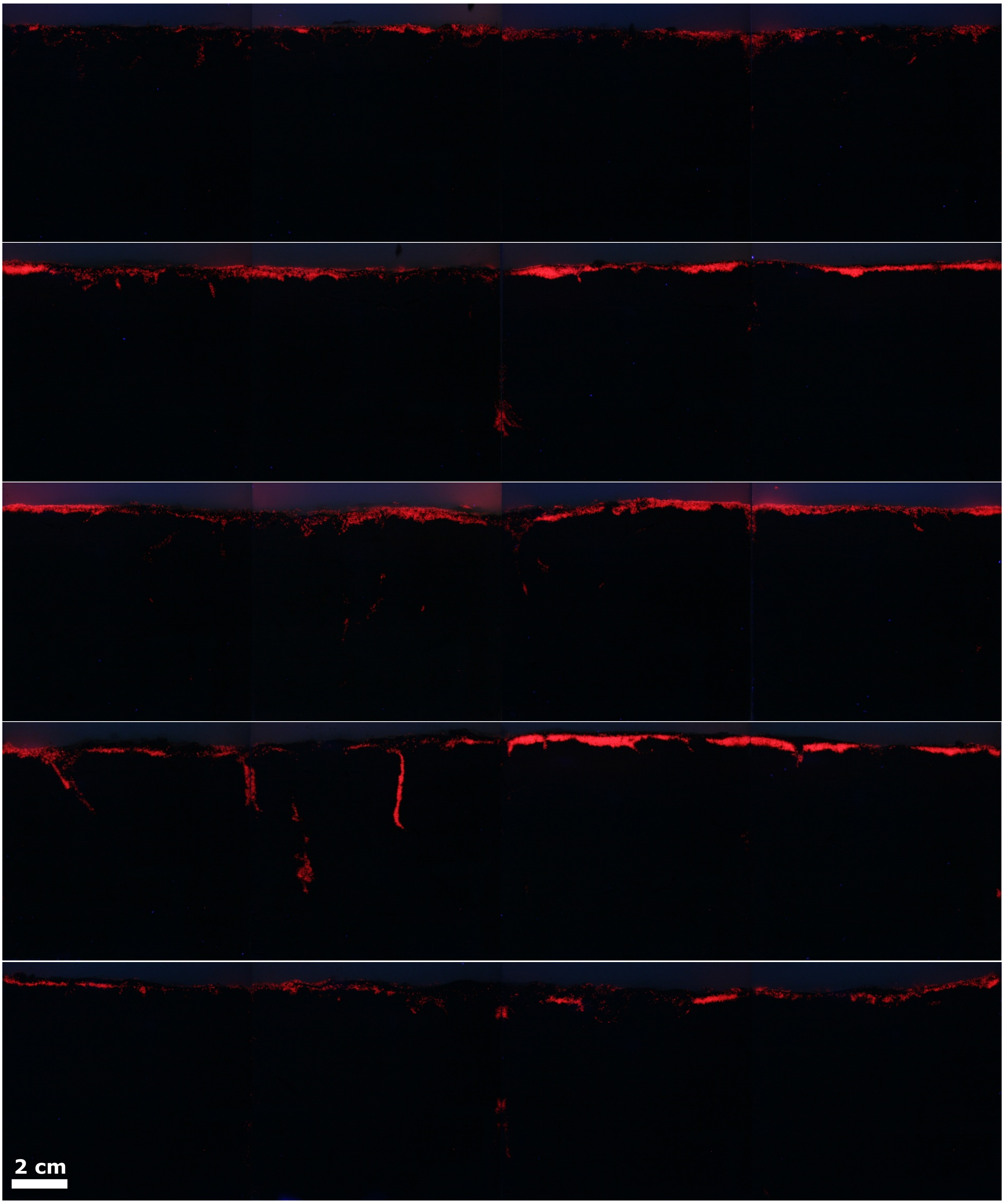

**Supplementary Figure S6 |** Sediment particle reworking profiles (n = 5) derived from the f-SPI images for (a) *Hediste diversicolor*, (b) *Hydrobia ulvae*, (c) *Corophium volutator*, and (d) in species mixture. Insets show detail of main figure.

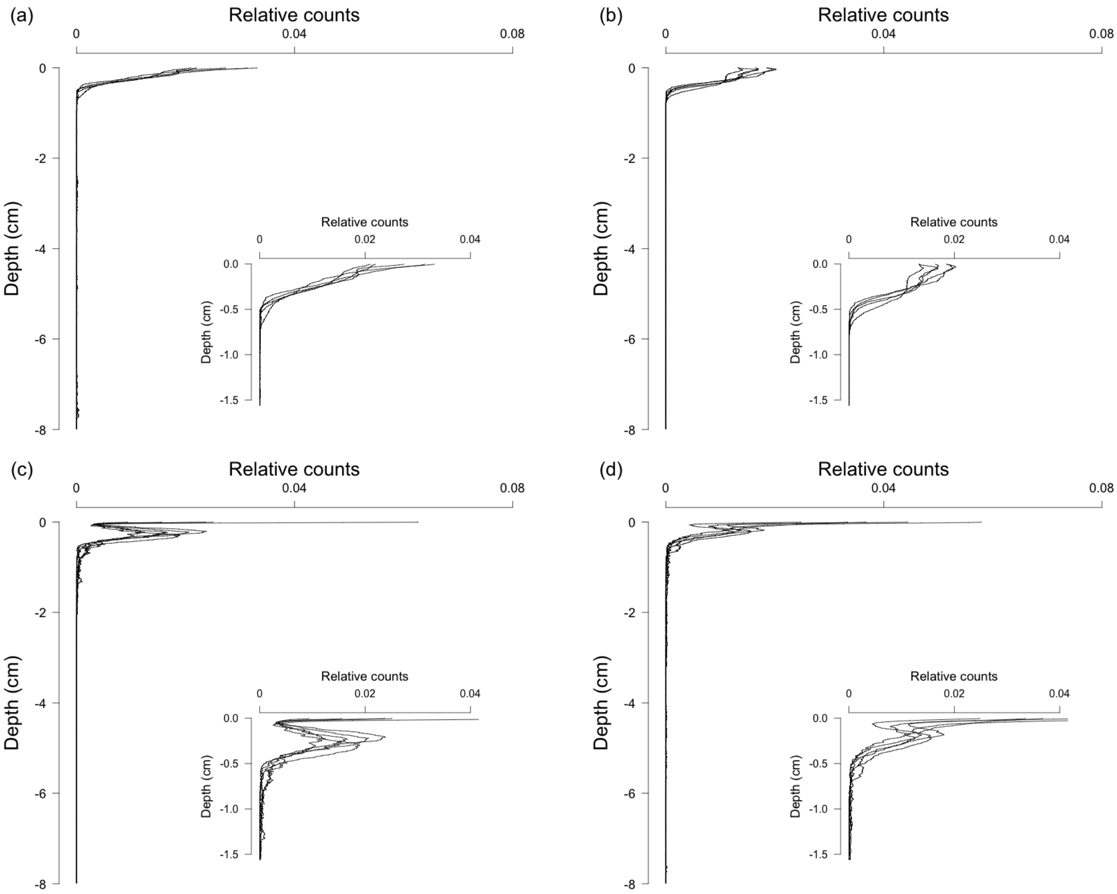

## Computed Tomography Images and movie sequences

Replicate (n = 5) cross sectional images were reconstructed for (i) the transverse plane 0.5 cm below the sediment-water interface (Suppl. Fig. S7) and (ii) the coronal plane through the rotational centre of the core (Suppl. Fig. S8). Three-dimensional models were created by segmentation of the burrows from the surrounding sediment (Suppl. Fig. S9).

**Supplementary Figure S7 |** Transverse core slices taken at 0.5 cm below the sediment-water interface for (a) *Hediste diversicolor*, (b) *Hydrobia ulvae*, (c) *Corophium volutator*, and (d) in species mixture. All cores are 10 cm in diameter. Burrows appear as darker grey values. In (b) and (d), the detail (e.g. aperture, whorls and apex) of *H. ulvae* shells can be seen (white pixel values).

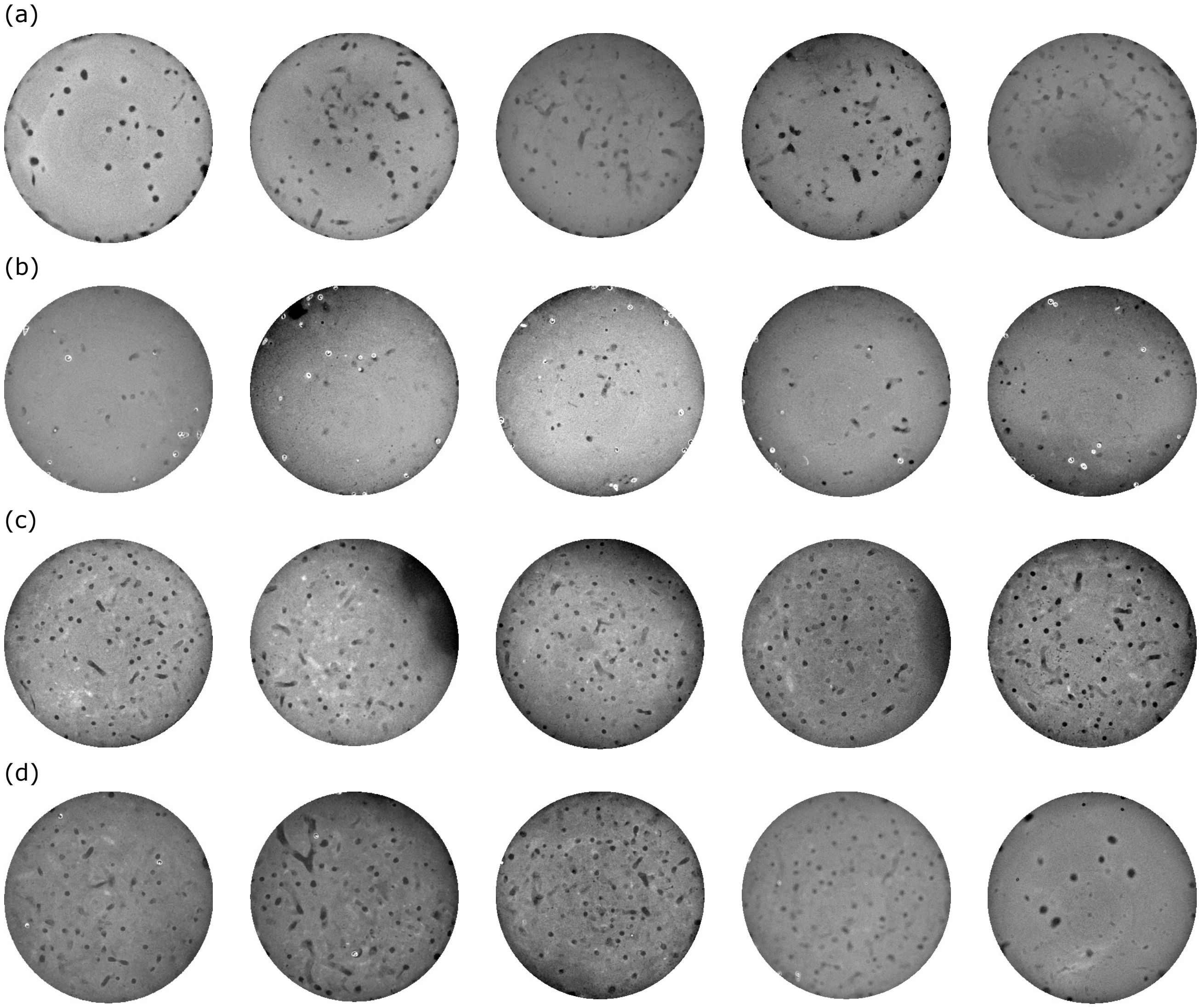

**Supplementary Figure S8 |** Replicate (n = 5) coronal core slices for (a) *Hediste diversicolor*, (b) *Hydrobia ulvae*, (c) *Corophium volutator*, and (d) in species mixture. Burrows appear as darker grey values. In (b) and (d), the detail (e.g. aperture, whorls and apex) of *H. ulvae* shells can be seen (white pixel values). The sediment-water interface is at the top of the region of interest. Images are cropped immediately below the vertical extent of burrowing. All images are 10 cm in width.

(a)

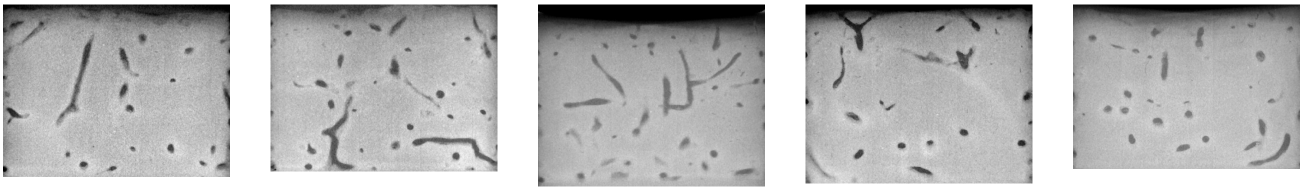

(b)

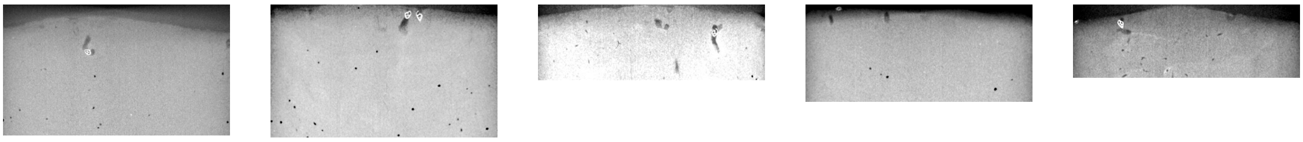

(c)

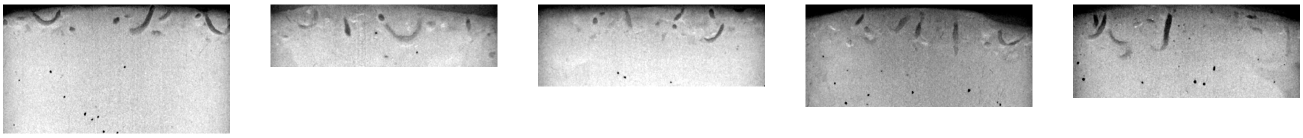

(d)

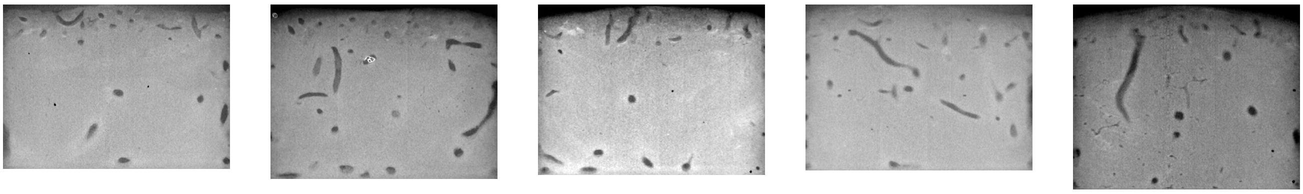

**Supplementary Figure S9 |** Reconstructed three-dimensional burrow models (n = 5) for (a) *Hediste diversicolor*, (b) *Hydrobia ulvae*, (c) *Corophium volutator*, and (d) in species mixture. In (b) and (d), the detail (e.g. aperture, whorls and apex) of *H. ulvae* shells can be seen (white pixel values). The sediment-water interface is at the top of the region of interest. Images are cropped immediately below the vertical extent of burrowing. All cores are 10 cm in diameter. All cores are 10 cm in diameter.

(a)

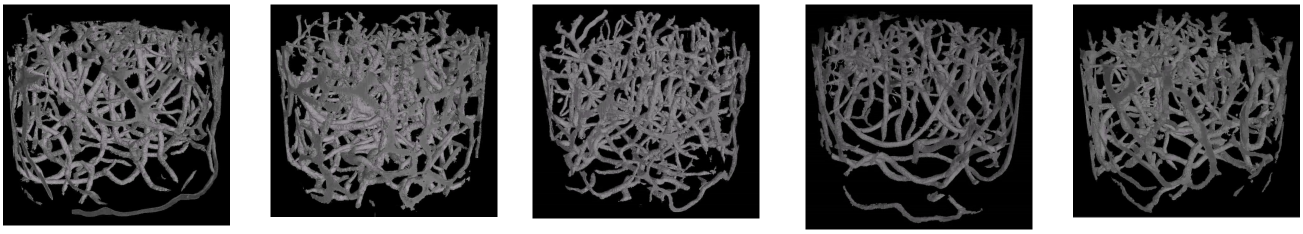

(b)

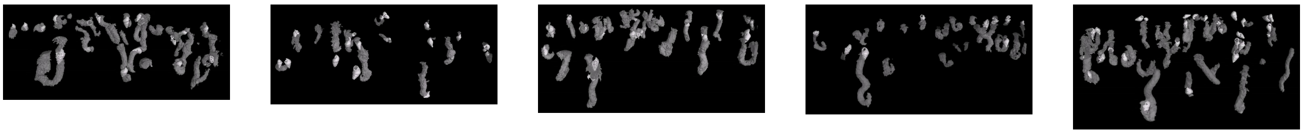

(c)

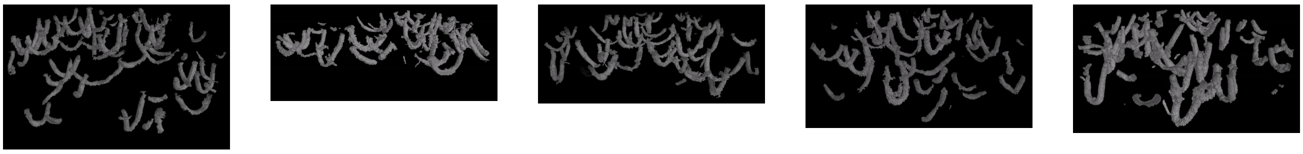

(d)

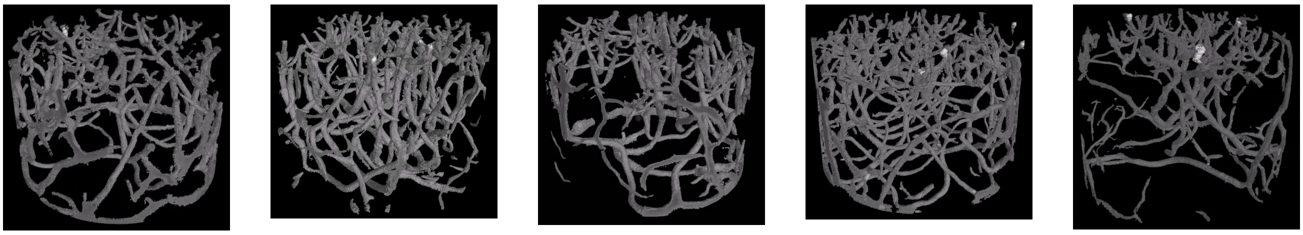

**Supplementary movie reconstructions of Figure S9** | Rotational three-dimensional movie reconstructions of each core replicate (n = 5) depicted in Suppl. Fig. S9 (above) are available online for *H. diversicolor* (**Suppl. Movie Sequence 1**), *H. ulvae* (**Suppl. Movie Sequence 2**), *C. volutator* (**Suppl. Movie Sequence 3**) and species in mixture (**Suppl. Movie Sequence 4**). Higher resolution movies (1920 × 720 pixels) are available on request from the corresponding author.

ENDS.
